# Supplementary material for: Active site specificity profiling datasets of matrix metalloproteinases (MMPs) 1, 2, 3, 7, 8, 9, 12, 13 and 14
Source: Data Brief. 2016 Feb 22;7:299–310. doi: 10.1016/j.dib.2016.02.036 (PMC4777984; doi:10.1016/j.dib.2016.02.036)
Supplement: Supplementary file 10 — Supplementary material [file mmc10.zip › WebPICS_hMMP9_G_1%/subsite_coop.html]

 

PICS results


- # About
- # Manual
- # Analysis
- # Results

- Redraw
- Seqlogo
- Dependency
- Coop

**Potential subsite cooperativity analysis for hMMP9\_G\_1%**   
Minimum difference for subsite dependency was set to +- 10 percentage-points  
Subsite dependency was checked for all positional occurences above 1 x natural abundance  
**Subsite dependency check is restricted to P3 - P3'**

|  |  |  |  |
| --- | --- | --- | --- |
| Fixed residue | Affected residue(s) | Change (percentage-points) | Vice-Versa change |
| P3\_A | P1\_A | 15.4 | 14.1 |
| P3\_A | P1prime\_I | 12.2 | 12.2 |
| P3\_A | P2prime\_L | 10.4 | 12.7 |
| P3\_A | P3prime\_G | 28.8 | 20.4 |
| P3\_A | P3prime\_S | -16.7 | -11.9 |
| P3\_V | P1prime\_Q | 14.9 | 16.1 |
| P3\_V | P2\_A | 25.5 | 17.4 |
| P2\_A | P1prime\_I | 16.5 | 14.3 |
| P2\_A | P2prime\_A | 12.2 | 17.8 |
| P2\_C | P2prime\_C | 97.3 | 48.6 |
| P2\_K | P1\_A | 13.6 | 11.3 |
| P2\_K | P1\_R | 14.7 | 19.6 |
| P2\_K | P2prime\_K | 12.7 | 14.1 |
| P2\_K | P2prime\_V | 16.3 | 16.3 |
| P2\_K | P3prime\_G | 18.8 | 12.1 |
| P2\_Q | P1\_Q | 12.6 | 12.6 |
| P2\_Q | P1prime\_Q | 25.1 | 12.6 |
| P2\_Q | P2prime\_R | 38.4 | 13.5 |
| P2\_R | P1prime\_V | 11.8 | 19.6 |
| P2\_Y | P1\_H | 16.6 | 16.6 |
| P2\_Y | P2prime\_Q | 15.9 | 13.3 |
| P1\_A | P1prime\_Q | 21.0 | 41.9 |
| P1\_A | P2prime\_K | 16.9 | 22.5 |
| P1\_C | P2prime\_Q | 45.9 | 15.3 |
| P1\_D | P1prime\_C | 13.9 | 17.7 |
| P1\_G | P1prime\_C | 11.5 | 22.0 |
| P1\_G | P1prime\_F | 13.5 | 35.6 |
| P1\_G | P2prime\_A | 14.9 | 24.1 |
| P1\_G | P2prime\_T | 17.6 | 41.2 |
| P1\_G | P3prime\_S | 21.7 | 14.6 |
| P1\_H | P2prime\_Q | 35.9 | 29.9 |
| P1\_K | P1prime\_I | 21.3 | 10.7 |
| P1\_K | P3prime\_G | 33.3 | 11.9 |
| P1\_N | P1prime\_F | 11.2 | 16.8 |
| P1\_N | P2prime\_I | 16.1 | 14.9 |
| P1\_N | P3prime\_N | 20.9 | 41.8 |
| P1\_Q | P3prime\_N | 12.6 | 12.6 |
| P1\_R | P2prime\_V | 26.3 | 19.7 |
| P1\_S | P2prime\_L | 12.7 | 11.2 |
| P1prime\_C | P2prime\_A | 18.4 | 15.6 |
| P1prime\_F | P2prime\_L | 25.2 | 11.2 |
| P1prime\_L | P3prime\_S | 10.6 | 15.1 |
| P1prime\_Q | P2prime\_K | 29.4 | 19.6 |
| P1prime\_Q | P2prime\_Q | 20.9 | 41.8 |
| P1prime\_V | P2prime\_I | 24.4 | 22.6 |
| P1prime\_V | P3prime\_N | 12.6 | 25.1 |
| P2prime\_I | P3prime\_N | 19.0 | 41.1 |
| P2prime\_V | P3prime\_A | 18.8 | 12.1 |

  
**PICS analysis of protease: hMMP9\_G\_1%**  
146 cleavage sites analyzed  
PICS library made with (T)rypsin, (G)luC or (C)hymotrypsin:   
Cutoff for graphic display: 2 x natural abundance  

|  |  |
| --- | --- |
| Positional occurences  (table for total and relative (in %) values) | Occurences relative to natural abundance (table) |
|  |  |
